# Inflammatory and Hemolytic Responses of Microaxial Flow Pump Temporary Ventricular Assist Devices via Axillary Access in Cardiogenic Shock

**DOI:** 10.3390/medicina60121960

**Published:** 2024-11-28

**Authors:** Leonie Schmack, Sadeq Ali-Hasan-Al-Saegh, Alexander Weymann, Nikolaus Pizanis, Payam Akhyari, Alina Zubarevich, Jasmin Sarah Hanke, Aron-Frederik Popov, Arjang Ruhparwar, Tienush Rassaf, Markus Kamler, Peter Luedike, Bastian Schmack

**Affiliations:** 1 Department of Cardiology and Vascular Medicine, West German Heart and Vascular Center Essen, University Hospital Essen, University Duisburg-Essen, 45141 Essen, Germany; leonie.schmack@krh.de (L.S.);; 2 Klinikum Region Hannover, Klinikum Siloah, Klinik für Kardiologie, Rhythmologie und Internistische Intensivmedizin, Stadionbrücke 4, 30459 Hannover, Germany; 3 Department of Cardiothoracic, Transplantation and Vascular Surgery, Hannover Medical School, Carl-Neuberg-Straße 1, 30625 Hannover, Germanyschmack.bastian@mh-hannover.de (B.S.); 4 Department of Thoracic and Cardiovascular Surgery, West German Heart and Vascular Center Essen, University Hospital Essen, University Duisburg-Essen, 45141 Essen, Germany; 5 Nils-Stensen-Kliniken, Marienhospital Osnabrück, Klinik für Innere Medizin, Kardiologie und Intensivmedizin, Bischofsstraße 1, 49074 Osnabrück, Germany

**Keywords:** Impella, ventricular assist devices, cardiogenic shock, extracorporeal membrane oxygenation

## Abstract

*Background and Objectives:* The use of temporary left ventricular assist devices (tLVADs) for patients suffering from cardiogenic shock (CS) is becoming more common. This study examines the indications and outcomes of microaxial flow pumps (Impella^®^, Abiomed Inc., Danvers, MA, USA) when cannulated through the axillary artery in patients with severe CS, with a particular focus on acute phase reactions and hemolytic responses. *Materials and Methods:* This single-center, retrospective cohort involved patients who received microaxial Impella implantation via the axillary artery from 2020 to 2022 (*n* = 47). *Results:* Among the patients, 66% (N = 31 cases) were treated with the Impella 5.5, 25.5% (N = 12 cases) with the Impella 5.0, and 8.5% (N = 4 cases) with the Impella CP. Additionally, 28% were managed using the ECMELLA concept. The mean length of time for Impella support was 8 days. The overall 30-day survival rate was 78%, with no significant differences observed between the ECMELLA group and the various Impella types. At 30 days post-therapy, 47% of survivors no longer required mechanical support, while 26% were upgraded to a durable LVAD. Interleukin-6 (IL-6) levels were significantly lower in patients receiving Impella 5.5 (*n* = 17 vs. 12) immediately following implantation (*p* = 0.03) compared with those with smaller devices. Haptoglobin levels were significantly higher in the Impella 5.5 group (*n* = 17 vs. 11, *p* = 0.02), with overall lower rates of hemolysis (45.1%, *p* < 0.01). *Conclusions:* The mortality rate in critical CS appears reduced with axillary artery implantation of Impella devices relative to existing literature. A full-flow microaxial pump (Impella 5.5) seems advantageous regarding systemic inflammatory response syndrome (SIRS) and acute hemolysis, indicated by lower IL-6 and higher haptoglobin levels, compared with smaller Impella devices. A tailored escalation/de-escalation concept using axillary access for different mAFP types appears feasible and safe.

## 1. Introduction

Cardiogenic shock (CS) is commonly defined as severe impairment of myocardial contractility resulting in diminished cardiac output, end-organ hypoperfusion and hypoxia [1]. Clinically, this condition manifests as hypotension unresponsive to volume resuscitation, along with signs of end-organ hypoperfusion [1]. Although new therapeutic options have emerged recently, management of CS with pharmacological or mechanical intervention is challenging and in-hospital mortality of CS remains high (30–60%), even if treated early [2]. Notably, the highest mortality occurs within the first 24 h of presentation, approaching 50% [3].

Temporary left ventricular assist devices (tLVAD) are increasingly applied as a therapeutic technique in patients with CS. Accordingly, the 2021 guidelines of the European Society of Cardiology (ESC) for treatment of acute heart failure patients has raised the recommendation level for short-term mechanical circulatory support (MCS) systems such as Impella heart pumps from Class IIb (“may be considered”) to Class IIa (“should be considered”) [4]. Specifically, microaxial flow pumps (mAFP, e.g., Impella^®^, Abiomed Inc.) are indicated for short- to mid-term therapy in CS stages C and D (SCAI classification) as a bridge to decision, bridge to recovery, or in high-risk coronary interventions, including protective cardiac surgeries like high-risk mitral valve procedures [5] or as part of a bridge-to-transplant strategy for VAD or transplant candidates [6,7,8].

Various types of Impella devices are available, including the Impella CP, Impella RP, Impella 5.0, and Impella 5.5. Impella RP is the only device designed for (partial) right ventricular (RV) support. For (partial) left ventricular support, Impella CP is preferred. Impella 5.0 and 5.5. are full-flow support devices with up to 5.5 L/min antegrade arterial blood flow. While the implantation of Impella CP and RP are completed percutaneously, for the implantation of the Impella 5.x devices, a surgical vascular access is mandatory. Impella 5.0 allows comparably longer support duration and differs from previous devices in device rigidity, shorter motor and lack of pigtail. A combination of an extracorporeal life support (ECLS) with a microaxial device for selective left ventricular unloading is referred to as the ECMELLA or ECPELLA concept [9,10].

The insertion and application of Impella devices into the blood stream triggers tissue damage and an unspecific immune reaction, known as the acute phase reaction or Systemic Inflammatory Response Syndrome (SIRS). Various mediators, including interleukin-1 and -6 (IL-6), and TNF-α stimulate the liver to synthesize acute phase proteins such as C-reactive protein (CRP), haptoglobin, and procalcitonin (PCT), peaking within the first 48 h post-implantation [11,12].

Moreover, hemolysis is a common complication in patients with tLVAD support lasting longer than 6 h, leading to issues such as anemia, jaundice, hemoglobinuria, and acute kidney injury [13]. Hemolysis is indicated by a sustained decline in hemoglobin and haptoglobin, alongside increased lactate dehydrogenase (LDH) and bilirubin levels, as well as a higher reticulocyte count. Among these, haptoglobin is considered the most sensitive marker, with a concentration below 0.2 g/L providing a diagnostic sensitivity of 83% and specificity of 96% for hemolysis, and an 87% probability of predicting hemolytic disease when serum haptoglobin falls below this threshold [14]. Consequently, laboratory markers such as haptoglobin, LDH, CRP, and IL-6, commonly used in intensive care settings, should be contextualized following MCS induction through Impella implantation.

This study aims to investigate outcomes and differentiate the inflammatory and hemolytic responses following axillary artery cannulation for various mAFP types in patients suffering from CS.

## 2. Materials and Methods

This study is a single-center, retrospective cohort that includes all patients meeting the criteria for CS as defined by the Shock classification of the Society for Cardiovascular Angiography and Interventions (SCAI C, D, and E) from 2020 to 2022. The enrolled patients in this study underwent mAFP implantation through the axillary artery. Cardiogenic shock was also classified using Interagency Registry for Mechanically Assisted Circulatory Support (INTERMACS) and SCAI classification. The implantation strategy was oriented to the previously proposed clinical flowchart [15]. We investigated patient characteristics at mAFP insertion and type of mAFP implanted or other MCS devices in place, such as ECMO, combined with mAFP treatment—defined as ECMELLA. We also studied the clinical assessment such as laboratory parameters, especially hemolytic indicators and laboratory signs for inflammation directly after implantation, 24 h after insertion and before device removal. We assessed available hemodynamics and adverse events on MCS. Follow up times were 30 days and 90 days after Impella implantation.

The study was approved by the local Ethics Committee of the University Duisburg-Essen (vote number 22-10BO).

Statistical analysis included sum, percentage and mean for numerical variables, Pearson’s chi-squared test for categorical data, paired *t*-test for continuous variables as well as Kaplan–Meier estimator for mortality. The level of significance was lower than 0.5. Statistical analysis was performed using IBM SPSS Statistics 28 and Microsoft Excel version 16.59.

## 3. Results

A total of 47 patients were identified and included in this study. Basic demographic characteristics at the time of mAFP implantation are summarized in Table 1. Among the patients, 32% presented with acute heart failure (*n* = 15), while 68% had acute or chronic decompensated cardiomyopathy (*n* = 32). Successful resuscitation was performed prior to Impella implantation in 32% of cases (*n* = 15). Detailed information about enrolled patients based on the different MCS systems is presented in Table 2.

At the time of implantation, 77% of patients exhibited kidney failure, with a mean creatinine level of 176 µmol/L, and 49% had liver failure, indicated by a bilirubin level of 4.90 mg/dL. Most patients (94%, *n* = 44) were dependent on inotropic support, and nearly all (98%, *n* = 46) required mechanical ventilation. The ejection fraction (EF) was below 40% in all patients (*n* = 47, 100%), averaging 19% and consistent with heart failure with reduced ejection fraction (HFrEF). Echocardiographic assessments revealed relevant mitral regurgitation in 31 patients (66%), tricuspid regurgitation in 22 (47%), and aortic regurgitation in 7 (15%).

**Figure 1:**
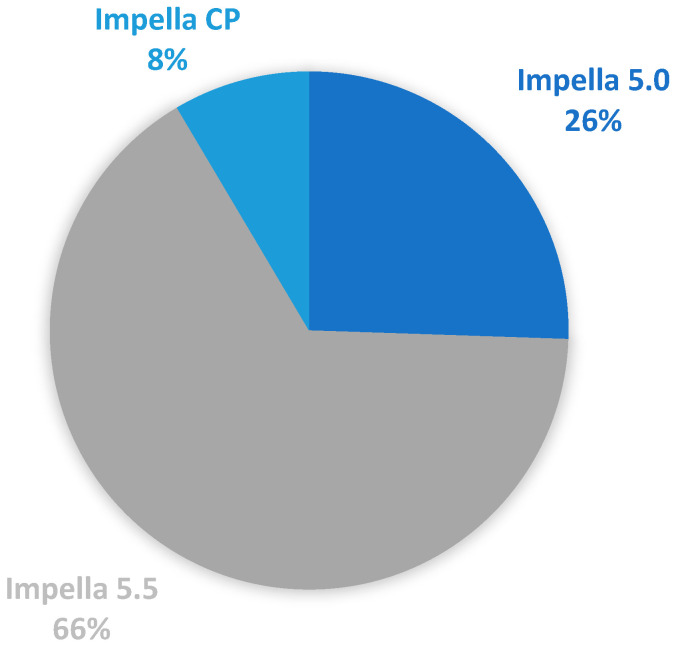
Different types of Impella devices for axillary artery implantation.

According to the INTERMACS classification, 57% of patients were at level 1, 36% at level 2, and 6.4% at level 3. The SCAI classification indicated that 53% of patients were classified as SCAI D or higher. Among the devices used, 66% received the Impella 5.5, 26% the Impella 5.0, and 8% the Impella CP (Figure 1).

**Figure 2:**
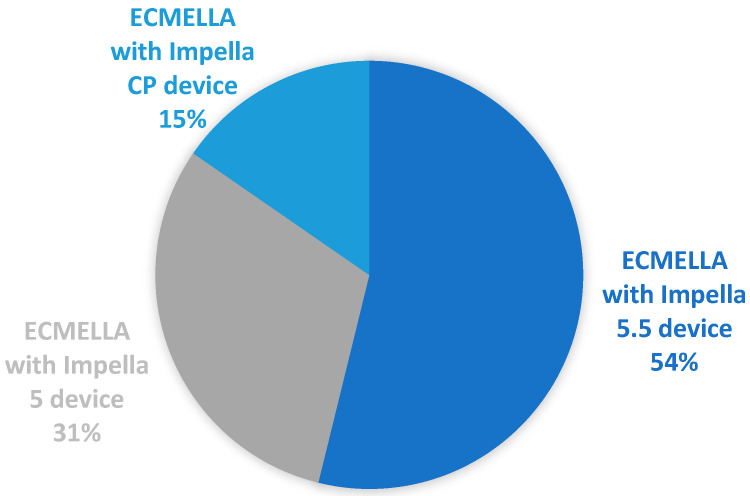
Different types of ECMELLA concept.

The primary conditions leading to cardiogenic shock included decompensated dilated cardiomyopathy (DCM) (*n* = 20, 42.5%), ischemic cardiomyopathy (*n* = 17, 36.1%), and post-cardiotomy cardiogenic shock (PCCS) (*n* = 12, 25.5%) (Table 1 and Table 2). A total of 46% of patients (*n* = 21) were initially treated with extracorporeal life support (ECLS). Of these, ECLS was removed at the time of Impella implantation in 8 patients (17%). The ECMELLA concept was utilized in 13 patients (28%), with 85% of the ECMELLA subgroup (*n* = 11) receiving ECLS prior to Impella insertion via axillary access (Figure 2).

The average duration of Impella support was 8 days, and the average follow-up time was 169 days. The 30-day survival rate for all Impella patients was 72.3%, with no significant differences between those treated with ECMELLA (69.2%) and those without (mAFP alone, 73.5%) or among different mAFP types. The overall 90-day survival rate was 70.2%. At 30 days post-initiation of mAFP therapy, 47% of patients no longer required any mechanical support, while 26% were transitioned to a durable left ventricular assist device (LVAD).

**Figure 3:**
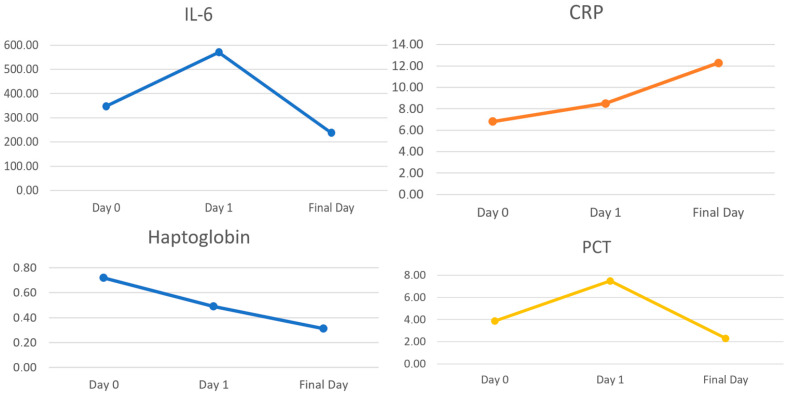
Changes in inflammatory and hemolytic factors (IL-6, CRP, haptoglobin, PCT).

**Figure 4:**
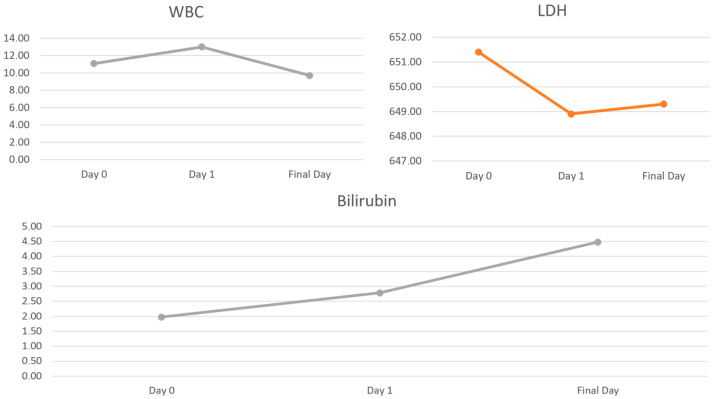
Changes in inflammatory and hemolytic factors (WBC, LDH and bilirubin).

IL-6 levels did not show a significant increase on the first day following mAFP implantation and normalized before device removal (347.6 pg/mL preoperatively vs. 570.7 pg/mL, *p* = 0.41; 238.2 pg/mL, *p* = 0.55, Table 3) (Figure 3). CRP levels significantly increased 24 h after Impella insertion (6.83 mg/dL vs. 8.50 mg/dL, *p* = 0.02) and continued to rise during Impella support (12.3 mg/dL, *p* < 0.01) (Figure 3). WBC counts also rose significantly on the first day (11.1 × 10^9^/L vs. 13.0 × 10^9^/L, *p* = 0.15) but had normalized by the last day (9.7 × 10^9^/L, *p* = 0.43) (Figure 4). PCT was elevated only on the first day after insertion (3.87 µg/L vs. 7.49 µg/L, *p* < 0.01) but normalized during Impella support (2.30 µg/L, *p* = 0.43, Table 3). Haptoglobin levels decreased significantly 24 h after insertion (0.72 g/L vs. 0.49 g/L, *p* = 0.01) and remained low (0.31 g/L, *p* = 0.03). No significant differences were observed in LDH levels (651.4 U/L vs. 648.9 U/L, *p* = 0.94; 649.3 U/L, *p* = 0.99). Bilirubin levels were significantly elevated (1.98 mg/dL vs. 2.80 mg/dL, *p* < 0.01; 4.45 mg/dL, *p* < 0.01, Table 3) (Figure 4).

Looking at subgroups, IL-6 was considerably lower in patients treated with Impella 5.5 in comparison with Impella 5.0 and CP (*n* = 17 vs. 12) immediately after implantation (*p* = 0.03), while no difference in CRP, PCT or WBC were detected. In addition, haptoglobin was significantly higher in Impella 5.5 (*n* = 17 vs. 11) during left ventricular support (*p* = 0.02) without distinction as to other hemolytic markers (LDH and bilirubin).

Overall, 20 patients (42.6%) required blood transfusions, with no significant differences among the mAFP subgroups (31.3% vs. 48.4%, *p* = 0.27). On the first day, hemolysis (defined as LDH > 240 U/L and haptoglobin < 0.2 g/L) was detected in 13 patients (27.7%). Hemolysis occurred in 12.9% of the Impella 5.5 group, in contrast to 56.3% of patients with smaller mAFP devices (*p* < 0.01). The cumulative incidence of hemolysis was 30 patients (63.8%), with a significantly higher incidence in the smaller Impella cohort (100% vs. 45.1%, *p* < 0.01).

Regarding adverse events, there was one case of pump thrombosis (Impella CP) and one case of device malfunction (Impella 5.5, not in ECMELLA), both leading to explantation for bridge to recovery. No cases of pump exchange or dislocation were reported. Vascular complications requiring surgical intervention occurred in two cases, but no limb ischemia was detected. Four patients experienced acute neurological events (8.67%), including one case of critical illness polyneuropathy (2.17%) and one patient requiring vacuum assisted closure (VAC) therapy due to axillary Impella insertion (2.17%). New-onset renal replacement therapy was required in 17 patients (37.10%), evenly distributed across groups.

## 4. Discussion

Impella has emerged as an increasingly used treatment option in CS. The ECLS-shock trial demonstrates that early ECLS does not improve survival in patients with AMI complicated by cardiogenic shock who are scheduled for early revascularization (30-day mortality 47.8% in the ECLS group and 49.0% in the control group) and is associated with an increase in complications [16]. Iannaccone et al. performed a meta-analysis and reported, in the form of forest plots of retrospective studies, that, despite the use of Impella, 30-day mortality in CS is still high (47.8%) [17]. Their meta-analysis of retrospective cohorts showed that the utilization of an Impella CP, initiation of Impella in patients without cardiac arrest and before PCI can be correlated with a better outcome. Our retrospective cohort shows an improved 30-day survival rate of 68% (30-day mortality of 32%) in patients with critical CS undergoing Impella implantation via axillary artery, which is better than previously reported outcomes in the meta-analysis by Iannaccone et al. [17]. Survival rates did not differ significantly among the various Impella devices used. Vascular complication rates were low, including re-operation and ischemia [18].

The surgical access approach towards the axillary artery offers advantages such as safe vascular access for anastomosing a vascular graft end-to-side to the artery, preventing distal malperfusion. Moreover, the enhanced mobilization options [18], which are particularly beneficial for patients requiring extended MCS (up to 29 days with the Impella 5.5), contribute to an improved functional outcome. The risk of stroke associated with Impella placement is a significant concern. In our cohort, the stroke rate was 6.38% (one patient with ECMELLA and two patients without ECMELLA) which is slightly lower than the 10.3% reported in existing literature [19]. Notably, the occurrence of adverse neurological events does not appear to be associated with the method of vascular access [19].

ECLS was primarily utilized prior to Impella implementation, allowing for the escalation of therapy to ECMELLA with sequential mAFP devices via axillary artery. In our study, the use of the ECMELLA concept did not lead to improved survival compared with mAFP alone. The concomitant treatment with venoarterial extracorporeal membrane oxygenation (VA-ECMO) and mAFP may enhance outcomes for patients with CS compared with VA-ECMO alone [20]. However, since an individualized escalation and de-escalation approach was applied [15], it can be hypothesized that patients receiving ECMELLA were more critically ill or clinically unstable, necessitating greater hemodynamic support.

As mentioned, various mAFP sizes and placement techniques are available. The Impella CP is typically inserted via the femoral artery. However, in cases involving ECMELLA or in cases when the axillary artery has a very small diameter or when severe peripheral vascular disease precludes femoral access, surgical access via the axillary artery becomes a viable alternative. Nonetheless, our data indicate that the Impella CP does not provide the same level of hemodynamic support as the designated full-mAFP (Impella 5.5).

Focusing on acute phase reaction, there was a rise, though not significant, in IL-6 levels as major regulator of the acute phase synthesis at day one after mAFP insertion. Consequently, the acute phase protein PCT peaked at the first day after mAFP insertion, while CRP remained elevated during support. Notably, IL-6 levels were significantly lower in patients treated with Impella 5.5 on the first day after device implantation, with no observed differences in CRP, PCT, or WBC counts. This leads to the cautious conclusion that the Impella 5.5 may induce a lesser acute phase reaction. IL-6 and related cytokines play crucial roles in the pathophysiology of heart failure [21]. Therefore, it can be inferred that the Impella 5.5 may inflict less harm while providing similar or even greater flow rates, suggesting that hemodynamics could be managed more sustainably. Hemolysis is a common complication in patients receiving mAFP support for CS. Previous studies, such as the Prospective Feasibility Trial Investigating the Use of the Impella 2.5 System in Patients Undergoing High-Risk Percutaneous Coronary Intervention (PROTECT I), reports hemolysis rates of 5% to 10% in the first 24 h after mAFP insertion [22]. The USpella registry has determined a hemolysis rate of 10.3% [23], while the EUROSHOCK registry indicates a rate of 7.5% among patients with an average support duration of 43 h [24].

In our cohort, the incidence of hemolysis on the first day was significantly higher in the smaller mAFP device subgroup (56.3%) but comparable to the literature for the Impella 5.5 (12.9%). Regarding overall hemolysis with slightly prolonged support (average of 8 days), the cumulative incidence of 63.8% aligns with previous findings (62.5%) [23]. Our results indicate that hemolysis was present from the first day of mAFP support and persisted until device removal. However, the Impella 5.5, being the largest device, appears advantageous, with significantly higher haptoglobin levels and clinically fewer cases of hemolysis (45.1%). This suggests a clear superiority of the Impella 5.5 concerning acute hemolysis. We hypothesize that reduced shear stress within the Impella 5.5 may contribute to these favorable outcomes.

In our study, the need for blood transfusions was similar across both subgroups, there were fewer transfusions overall in patients receiving Impella via axillary artery access (42.6%) compared with those with traditional access (65%) as noted in the literature [23]. It is important to consider that blood transfusions expose transplant candidates to various antigens, potentially elevating panel-reactive antibodies. Thus, surgical access via the axillary artery may offer additional benefits. However, as smaller mAFP devices did not differ significantly in other hemolytic markers, the requirement for blood transfusions, acute phase reaction markers (except for IL-6), or survival rates, it cannot be conclusively stated that “bigger is better”. We conclude that there is a valid indication for the use of Impella CP via the axillary artery, particularly when the Impella 5.5 may be too large for the vessel diameter, for venting purposes, or when other factors render its use unfeasible. In all cases involving mAFP devices, monitoring hemolysis parameters at baseline and at frequent intervals is essential.

Our study is limited by the small number of participants in each subgroup and its retrospective nature, which prevents us from drawing definitive conclusions. Additionally, with 38.3% of patients requiring renal replacement therapy and 30.8% treated with the ECMELLA concept, the outcomes of our study may be confounded. It should be noted that our study has other limitations regarding the comparison of the systemic inflammatory response during cardiogenic shock based solely on the type of mechanical circulatory support employed. This approach may not adequately reflect the complexity of the condition. In particular, stages D and E of CS, as defined by the SCAI, are associated with immunoparalysis, which may influence the inflammatory response and should be considered in future analyses. In fact, randomized studies are needed to validate the interesting results, ideally including more detailed examinations of different Impella devices and of the ECMELLA 2.0 concept. Future research should also provide detailed hemodynamic data of interest, such as flow rates, cardiac index, pulmonary capillary wedge pressure, systemic vascular resistance, pulmonary vascular resistance, and central venous oxygen saturation, both with and without the Impella device.

## 5. Conclusions

Taking everything into account, our results indicate that the approach of utilizing an mAFP device via the axillary artery is both safe and practicable across all available Impella sizes. Notably, the data regarding IL-6 suggest potential benefits of using the larger Impella 5.5, which may be associated with reduced systemic inflammatory response syndrome and possibly better hemodynamic support.

Furthermore, to our knowledge this is the first time the incidence of hemolysis in prolonged Impella therapy via axillary artery has been quantified. In this setting, our study highlights a significantly higher incidence of hemolysis in patients with CS undergoing percutaneous hemodynamic support with smaller Impella devices compared with the larger Impella 5.5. However, this should also be considered a limitation for definitive conclusions, as the benefits of using the larger Impella 5.5 can only be accurately assessed by comparing patients with and without this device, taking into account pre-procedural characteristics, laboratory values, and outcomes in larger sample sizes.

Our findings suggest that the suspected higher amount of hemolysis occurring with smaller devices does not affect overall short-term outcomes or requirements of blood transfusion. Therefore, a tailored escalation/de-escalation support concept should be strived for in cases of CS as a successful bridge for definitive therapy or recovery.

## Figures and Tables

**Table 1:** Patient baseline characteristics at Impella implantation.

	Number	Percent
Number	47	-
Age (years)	58	-
Male	40	85%
Etiology of cardiogenic shock		
ST-elevation myocardial infarction	5	10.6%
Non-ST-elevation myocardial infarction	10	21.2%
Post cardiotomy cardiogenic shock (PCCS)	12	25.5%
Decompensated dilated cardiomyopathy (DCM)	20	42.5%
Decompensated ischemic cardiomyopathy (ICM)	17	36.1%
Decompensated valvular cardiomyopathy (VCM)	1	2%
Tachycardia-induced cardiomyopathy	4	9%
Myocarditis	3	6%
Cardiopulmonary resuscitation before insertion	15	32%
Left ventricular ejection fraction		
30–<40%	8	17%
20–<30%	20	43%
<20%	19	40%
Peripheral artery disease	4	8%
Cervical artery disease	8	17%
Hypertension	33	70%
Arterial fibrillation	23	49%
Diabetes mellitus	15	32%
COPD	5	11%

**Table 2:** Patient baseline characteristics based on the different MCS systems.

	Impella 5	Impella 5.5	Impella CP
Number	12 (25.5%)	31 (66%)	4 (8.5%)
Age (years)	61	58	56
Male	10 (83.3%)	27 (87%)	3 (75%)
Etiology of cardiogenic shock			
ST-elevation myocardial infarction	1 (8.3%)	4 (12.9%)	0 (0%)
Non-ST-elevation myocardial infarction	1 (8.3%)	8 (25.8%)	1 (25%)
Post cardiotomy cardiogenic shock (PCCS)	2 (16.6%)	9 (29%)	1 (25%)
Decompensated dilated cardiomyopathy (DCM)	6 (50%)	12 (38.7%)	2 (50%)
Decompensated ischemic cardiomyopathy (ICM)	2 (16.6%)	14 (45.1%)	1 (25%)
Decompensated valvular cardiomyopathy (VCM)	1 (8.3%)	0 (0%)	0 (0%)
Tachycardia-induced cardiomyopathy	0 (0%)	3 (9.6%)	1 (25%)
Myocarditis	1 (8.3%)	2 (6.4%)	0 (0%)
Cardiopulmonary resuscitation before insertion	3 (25%)	10 (32.2%)	2 (50%)
Left ventricular ejection fraction			
30–<40%	0 (0%)	7 (22.5%)	1 (25%)
20–<30%	9 (75%)	9 (29%)	2 (50%)
<20%	3 (25%)	15 (48.3%)	1 (25%)
Peripheral artery disease	2 (16.6%)	1 (3.2%)	1 (25%)
Cervical artery disease	4 (33.3%)	2 (6.4%)	2 (50%)
Hypertension	8 (66.6%)	21 (67.7%)	4 (100%)
Arterial fibrillation	5 (41.6%)	18 (58%)	0 (0%)
Diabetes mellitus	5 (41.6%)	8 (25.8%)	2 (50%)
COPD	1 (8.3%)	2 (6.4%)	2 (50%)

**Table 3:** Changes in inflammatory and hemolytic factors.

	Preoperation	First Day After Impella Implantation	During Impella Support
CRP (mg/dL)	6.83	8.50	12.3
IL-6 (pg/mL)	347.6	570.7	238.2
WBC (×10^9^/L)	11.1	13.0	9.7
PCT (µg/L)	3.87	7.49	2.30
Haptoglobin (g/L)	0.72	0.49	0.31
LDH (U/L)	651.4	648.9	649.3
Bilirubin (mg/dL)	1.98	2.80	4.45

## Data Availability

The data are available from the corresponding author upon reasonable request.
